# Motor training programs of arm and hand in patients with MS according to different levels of the ICF: a systematic review

**DOI:** 10.1186/1471-2377-12-49

**Published:** 2012-07-02

**Authors:** Annemie IF Spooren, Annick AA Timmermans, Henk AM Seelen

**Affiliations:** 1 Department of Healthcare, PHL University College Hasselt, Hasselt, Belgium; 2 Adelante Centre of Expertise in Rehabilitation and Audiology, Hoensbroek, The Netherlands; 3 Department of Rehabilitation Medicine, Maastricht University, Research School CAPHRi, Maastricht, The Netherlands; 4 Guffenslaan 39, 3500, Hasselt, Belgium

**Keywords:** Multiple sclerosis, Upper extremity, Rehabilitation, ICF, Training

## Abstract

**Background:**

The upper extremity plays an important role in daily functioning of patients with Multiple Sclerosis (MS) and strongly influences their quality of life. However, an explicit overview of arm-hand training programs is lacking. The present review aims to investigate the training components and the outcome of motor training programs for arm and hand in MS.

**Methods:**

A computerized systematic literature search in 5 databases (PubMed, CINAHL, EMBASE, PEDro and Cochrane) was performed using the following Mesh terms: Multiple Sclerosis, Rehabilitation, Physical Education and Training, Exercise, Patient-Centered Care, Upper Extremity, Activities of Daily Living, Motor Skills, Motor Activity, Intervention Studies and Clinical Trial. The methodological quality of the selected articles was scored with the Van Tulder Checklist. A descriptive analyses was performed using the PICO principle, including scoring of training components with the calculation of Hedges’g effect sizes.

**Results:**

Eleven studies were eligible (mean Van Tulder-score = 10.82(SD2.96)). Most studies reported a specific improvement in arm hand performance at the ICF level that was trained at. The mean number of training components was 5.5(SD2.8) and a significant correlation (r = 0.67; p < 0.05) between the number of training components and effect sizes was found. The components ‘client-centered’ and ‘functional movement’ were most frequently used, whereas ‘distribution based practice’, ‘feedback’ and ‘random practice’ were never used. The component ‘exercise progression’ was only used in studies with single ICF body function training, with the exception of 1 study with activity level training. Studies including the component ‘client-centred’ demonstrated moderate to high effect sizes.

**Conclusion:**

Motor training programs (both at the ICF body function and activity level) have shown to improve arm and hand performance in MS in which the value of the training specificity was emphasized. To optimize upper extremity training in MS the component ‘client-centred’ and ‘exercise progression’ may be important. Furthermore, given the importance attributed to the components ‘distribution based practice’, ‘feedback’ and ‘random practice’ in previous research in stroke patients, the use of these components in arm hand training should be explored in future research.

## Background

Multiple Sclerosis (MS) is a chronic disease of the central nervous system which is characterised by a demyelinisation and axonal loss within the central nervous system. This results in a loss of motor, sensory, cognitive and autonomic functions.

Dysfunctions of the lower extremities, causing a decline of walking capacity, are reported in 75% of persons with MS, whereas dysfunctions of the upper extremities occur in at least 66% of the persons with MS [1]. The level of arm and hand functioning determines for a great part the level of independence in daily activities like eating, dressing, grooming [2]. Johansson et al. reported that 76% of persons with MS experienced problems with manual dexterity [1] and 44% experienced problems with activities of daily living. Former problems may, in turn, influence the level of participation and quality of life [2].

Despite the importance of the upper extremity rehabilitation and the amount of clinical experience in this domain, limited research is dedicated specifically to upper extremity performance and training in persons with MS.

In general, the rehabilitation of the upper extremity in patients with neurological diseases is gaining interest. For spinal cord injury (SCI), Spooren et al. reviewed training programs for the upper extremity, demonstrating the limited number of studies in this domain [3]. They reported the benefits of motor training programs to improve upper extremity functioning and the importance of the specificity of the training. Due to the combination of a wide variety of upper limb activities and the importance of the specificity of the training, they suggested that a client-centred approach would be most beneficial [3]. For stroke patients, Van Peppen et al. reviewed the evidence for physical therapy interventions aimed at improving functional outcome [4]. With regard to upper extremity training, they concluded that there is strong evidence that patients benefit from exercise programmes in which functional tasks are directly and intensively trained [4]. Timmermans et al. reviewed the task-oriented upper limb training in stroke [5]. They distinguished different training components within a task-oriented training and concluded that the components ‘feedback’ and ‘distribution-based practice’ were associated with higher effect sizes post intervention and ‘random practice’ and ‘clear functional goal’ with largest effect sizes at follow-up.

Patients with MS may have both spinal cord and brain lesions. In contrast to patients with SCI and stroke, they show temporal fluctuations in impairment which makes direct translation from findings regarding training of the upper extremity in SCI and stroke difficult. The exacerbation and the fatigue have implications on the rehabilitation management of persons with MS [6,7]. Another difference with stroke patients is that MS patients may demonstrate uni- or bimanual impairment in which unimanual exercise programs are not always applicable to MS patients. . Like stroke and SCI, MS is a in chronic illness leading to patients encountering different needs throughout their lifespan [8]. The changing needs will be even more pronounced in MS patients, as MS is a progressive disease, in contrast to stroke and SCI [6].

Unlike for SCI and stroke, an overview of training programs of the upper extremity in MS is to the authors’ the knowledge, not yet available. Earlier research in MS focussed on the effectiveness of exercise therapy in terms of activities of daily living and health related Quality of Life [9] and on the effectiveness of multidisciplinary rehabilitation in different settings [10]. Steultjes et al. and Baker et al. reviewed the effectiveness of occupational therapy-related treatment, including a broad range of interventions such as physical, psychological and functional interventions [11,12]. Dalgas et al. and Wiles et al. focused on physiotherapy-related treatment of persons with MS [7,13]. Dalgas et al. provided recommendations for the application of resistance, endurance and combined training [7]. Wiles et al. noted that, although most physical therapy interventions result in an improvement in function or a better feeling, it is not clear which component of physical therapy is responsible for the improvement, as they have hardly ever been specified clearly [13]. Despite the fluctuations in impairment, findings of the above mentioned studies indicate that MS patients may benefit from motor training programs. However, an overview of upper extremities training programs and knowledge on the content of the training programs is lacking.

Given 1) the importance of the upper extremity in daily functioning of MS patients and 2) the lack of an explicit overview and content of upper extremity interventions in MS treatment, the current review aims to investigate the outcome of motor training programs for arm and hand functioning (on the ICF body function and activity level) in persons with MS, and the training components used in such programs.

## Methods

### Search strategy

A computerized search was conducted for all English, French, German and Dutch articles in Medline (Pubmed), Cochrane databases, Cinahl, Embase, PEDro and DARE. Studies were collected from 1976 up to May 2011. Reference lists of these articles and narrative reviews were also scanned for relevant publications.

Following Medical Subject Headings (MeSH) were used: ("Multiple Sclerosis”) AND (“Rehabilitation” OR “Physical Education and Training” OR “Exercise" OR “Physical Therapy Modalities" OR "Patient-Centered Care" OR “non-directive therapy”) AND ("Upper Extremity" OR "Activities of Daily Living" OR "Motor Skills" OR "Motor Activity” NOT "Lower Extremities" NOT "Walking") AND ("Intervention Studies" OR "Clinical Trial OR "Review Literature”).

### Eligible studies

Studies were included when persons with MS (at least 5 persons) were involved in an intervention study or a clinical trial in which a motor training program was used aimed at improving arm hand at the ICF body function or the ICF activity level and in which outcome was described on the ICF body function level or the ICF activity level.

### Ineligible studies

Studies on functional electro-stimulation, neuroprostheses or surgery were excluded. Studies featuring orthoses or assistive devices, and physical fitness studies focussing on physical capacity outcome or cardio-respiratory functioning were also excluded. Although the use of new rehabilitation techniques, such as the use of robotics or virtual reality, to improve arm and hand functioning is increasing and results seems promising [14], the present review did not include studies with robotic-assisted training programs. The study aimed to investigate training components. The use of robotics and assistive technology is considered a tool and not a training method or component as such.

Two independent observers conducted the data selection/extraction.

### Methodological assessment

Two independent observers (SA and TA) rated the methodological quality of the selected studies with the Van Tulder’s Quality assessment system [15]. This scale scores the internal validity (maximum 11 points), the descriptive criteria (maximum 6 points) and the statistical criteria (maximum 2 points) of RCT’s, but it can also be used to scale controlled clinical trials [15]. The internal validity criteria refer to characteristics of the study that might be related to selection bias, performance bias, attrition bias and detection bias, and should be used to define methodological quality in the meta-analysis. The descriptive criteria refer to the external validity of the study. The statistical criteria indicate whether calculations can be made and conclusion can be drawn independently of the opinion of the authors of the original study [15]. The interrater reliability of the individual items was tested using the Cohen’s Kappa [16]. The quality total Van Tulder score was obtained using the consensus method, i.e. the total score was calculated, after any disagreement on item scores was discussed and resolved.

### Descriptive assessment

The 2 independent observers analysed all the selected articles on the following items using the PICO principle (Patients, Intervention, Comparison and Outcome&Results) [17]. Subsequently, they scored each training on intervention components analogous to the study of Timmermans et al. 2010 [5]. Because the present review also included body function level training and non-task-oriented activity training, following training components were added: strength, endurance, mobility, basic activities of arm and hand (like grasping, moving objects) and complex activities (in which the whole body is involved). Detailed description of the components can be found in Table 1.

**Table 1:** **Description training components extended from Timmermans et al. 2010**[5]

Strength	Exercises following the ‘high resistance and low repetition’ rule [18,19]
Endurance	Exercises following the ‘low resistance and high repetition’ rule [18,19]
Mobility/stretching	Exercises aimed at improving range of motion
Basic activities	Activities of arm or hand like grasping, moving objects
Complex activities	Movement in which whole body is involved
Functional movement	a movement involving task execution that is not directed towards a clear ADL-goal (e.g. moving blocks from one location to another, stacking rings over a cone) (as opposed to analytical movements, which are movements without a goal, usually occurring in one single movement plane and often occurring in single joints, e.g. shoulder flexion)
Clear functional goal	a goal that is set during everyday-life activities, hobbies (e.g. washing dishes, grooming activity, dressing oneself, playing golf)
Client-centred	therapy goals that are set through the involvement of the patient him/herself in the therapy goal decision process. The goals respect patient’s values, preferences, expressed needs and recognize the clients’ experience and knowledge
Overload/overlearning	training that exceeds the patient’s metabolic muscle capacity(overload) or the performance needed for handling in daily life (overlearning) . Overload is determined by the total time spent on therapeutic activity, the number of repetitions, the difficulty of the activity in terms of coordination, muscle activity type and resistance load, and the intensity, i.e. number of repetitions per time unit. In this review we have scored a high amount of repetitions as determining factor for the presence of overload, as the other factors are rarely described in intervention descriptions.
Real object	manipulation that makes use of objects that are handled in normal everyday-life activities (e.g. cutlery, hairbrush…).
Context specific	a training environment (supporting surface, objects, people, room,…) that equals or mimics the natural environment for a specific task execution, in order to include task characteristic sensory/perceptual information, task specific context characteristics and cognitive processes involved
Exercise progression	Exercises on offer have an increasing difficulty level that is in line with the increasing abilities of the patient, in order to keep the demands of the exercises and challenges optimal for motor learning
Exercise variety	A variety of exercises was offered to support motor skill learning of a certain task because of the person experiencing different movement and context characteristics (within task variety) and problem solving strategies
Feedback	specific information on the patient’s motor performance that enhances motor learning and positively influences patient motivation (for more information, the authors refer to)
Multiple movement planes	Movement that uses more than one degree of freedom of a joint, therefore occurring around multiple joint axes.
Total skill	The skill is practiced in total, with or without preceding skill component training (e.g. via chaining)
Customized training load	A training load that suits the individualized treatment targets (e.g. endurance, coordination or strength training) as well as the patient’s capabilities (e.g. 65% of 1 repetition maximum or 85% of 1 repetition maximum for the specific patient).
Random practice	Each practice session, the exercises are randomly ordered
Distributed practice	A practice schedule with relatively long rest periods
Bimanual tasks	Tasks where both arms and hands are involved are included

If no consensus on the data extraction, on the methodological and on the descriptive assessment was achieved between the 2 independent observers, a third independent observer made the final decision.

To describe the training effects of the studies included, both level of significance and effect sizes (ES) statistics was used. For the latter, where possible, the “Hedges’ g” was established by calculating the difference between means of the baseline values and the post-intervention values divided by the pooled standard deviation. According the classification of Cohen, ES < 0.2 were considered as small, from 0.2 -0.5 as moderate and > 0.5 as large [16,20]. If no means and standard deviations of the raw data were available, authors were contacted or the effect sizes were used that were reported by the authors themselves (in which it should be noted that these were not always the “Hedges’ g”). A correlation coefficient (Spearman’s rho) was calculated between the number of training components that were used in the studies and the effect sizes that were reported. In case of multiple measurement instruments, the outcome measure providing the largest effect size was used.

## Results

**Figure 1:**
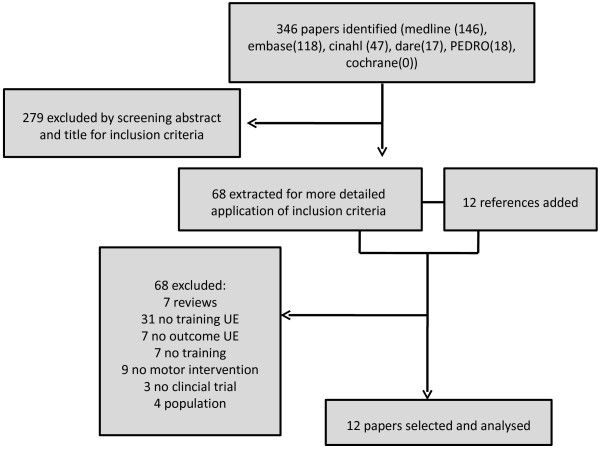
Flowchart paper selection.

Eleven studies were eligible and were included in the present review. Figure 1 presents the selection of the studies. Two papers of Romberg et al. 2004 and 2005 were eligible [21,22]. Because both papers reported the same intervention with different outcome measures, they were considered as 1 study in which the outcome of both papers was included.

### Methodological quality assessment

Table 2 demonstrates the total Van Tulder score, the subscores for internal validity, descriptive and statistics and the level of evidence according to the Dutch CBO (Central Guidance Organisation for Quality in Healthcare) (see Additional file 1: Appendix A) guidelines for each study. The mean Van Tulder score (using the consensus method) of the 11 included studies was 10.8 (SD 2.9) with a mean internal validity score of 4.5 (SD 2.1), a mean descriptive score of 4.4 (SD 0.9), a mean statistical score of 1.9 (SD2.9). The kappa score was 0.7 (SD 0.2) between the two raters, which was considered a substantial agreement according to Landis and Koch [23]. More details on the Van Tulder scoring are shown in Additional file 2: Appendix B.

**Table 2:** Van Tulder Score and patient characteristics

**Reference**	**Tulder**				**LOE**	**Design**	**Patients**							**Interv CG**
	**int val**	**descr**	**stat**	**total**			**MS type**	**EDSS**	**Age**	**TSFS**	**TSLE at least**	**EG (n)**	**CG (n)**	
Gehlsen et al. [24]	2	3	2	7	C	CS	?	?	40.2	?	?	10	no	
Romberg et al. [21,22]	5	6	2	13	B	RCT	?	1-5.5	44 (7.1)	6 (0–23)	1 mo	47	48	no
Taylor al. [25]	5	4	2	11	C	CS	?	0-6.5	45.6 (27–61)	6 (1–13)*	1 mo	12	no	
Freeman et al. [26]	4	4	2	10	B	RCT	SP,PP	5-9.5	43 (25–73)	15.4 (3–32)	1 mo	32	34	WL
Khan et al. [33]	9	5	2	16	A2	RCT	RR, SP,PP	0-8	49.5 (30–63)	10.7 (6.3)*	3 mo	48	50	WL (maint)
Mark et al. [27]	3	4	2	9	C	Pilot	PP, SP	6-7	56 (50–60)	?	3 mo	5	no	
Patti et al. [28]	7	6	2	15	A2	RCT	SP,PP	4-8	25-60	1.5 (0.5-2.5)	3 mo	58	53	home exerc.
Jones et al. [29]	2	4	1	7	B	CCT	moderate to severe ataxia and PP, SP	?	36.9 (8.2)	?	1 year	28	9	WL
Mathiowetz et al. [30]	3	4	2	9	C	CS	B, RR,PP, SP	2.5-8	45 (27–63)	12 (1–27)	?	30	no	
Storr et al. [31]	6	4	2	12	B	RCT	P, S, RR	0-9	53 (33–66)	15 (3–45)	3 mo	38	52	WL
Vikman et al. [32]	4	4	2	10	C	CS/CCT**	RR,SP,PP	4-6.5	55.8 (10.3)	18.3 (2–43)	?	40 + 18	18	WL

**B** (Benign); **CG** (Control Group); **CS** (Case Series); **CCT** (Controlled Clinical Trial); **Descr** (Descriptive score); **EDSS** (Expanded Disability Status Scale); **EG** (Experimental Group**); Interv CG** (Intervention Control Group); **Int Val** (internal Validity); **LOE** (Level of Evidence); **P** (Progressive); maint. (maintenance); mo (months) **PP** (Primary Progressive); **RR** (Relapse Remitting); **RCT** (Randomised Controlled Trial); **S** (Secondary); **SP** (Secondary Progressive); **TSFS** (time Since First Symptoms); **TSLE** (time since last exacerbation); **WL** (Waiting List); ? (not specified); *(Time since diagnosis); **(CCT for 18 persons).

Five of the 11 included studies were RCT’s [22,26,28,31,33], but only two of them [28,33] were double blinded and obtained an A2 level of evidence according to the Dutch CBO (Central Guidance Organisation for Quality in Healthcare) guidelines (see Additional file 1: Appendix A) [34].

### Descriptive analysis

#### Patients

Table 2 presents the patient characteristics of the studies included. A total of 368 persons with MS were included in the intervention groups of the studies. There was a large diversity in age (ranging between 25 and 73 years), MS severity (with an EDSS score [35] ranging from 0 to 9.5) and in the time since the occurrence of the first symptoms (ranging from 0–45 years). Time since the last exacerbation ranged from at least 1 month to 1 year in most of the studies included. The sample size of the intervention groups varied between 5 and 58 persons.

#### Intervention

Table 3 displays the details of the interventions regarding the upper extremity training. A wide variety regarding training content, duration (1–26 weeks), intensity and setting of the training between interventions was seen.

**Table 3:** Descriptive analysis

	**Set**	**Intervention**						**Outcome measures**		**Results**
**References**		**ICF level**	**Content**	**Weeks**	**Freq/ week**	**Freq/day**	**Intensity ('/day)**	**ICF level**	**Motor outcome UE**	
Gehlsen et al. [24]	out	FU	aquatic exercise (including freestyle swimming)	10	3	1	60 (60- 75% max heart rate)	FU	peak force, work, power (fatigue)	*improvement in force (ES:0.94), power(ES:0.79) and total work(ES:0.72); (not on fatigue (ES:0.23))
Romberg et al. [21,22]	in/out	FU	exercise program: strenght + aerobic	26	3-4 (resistance); 1(endurance)	?	?	FU + ACT	FU:UE endurance; ACT:FIM, MSFC (including 9HPT); BBT	*difference between groups on UE endurance, MSFC *(ES:0.1)*(mainly due to mobility factor); (not on BBT; FIM *(ES:0.15)*)
Taylor al. [25]	out	FU	PRE: UE (3ex)(and LE (3ex)):2x10-12rep	10	2	1	60	FU + ACT	FU:Arm press (1RM and endurance)	*improvement on arm press 1RM (ES:0.31); not on arm endurance (ES:0.47) (* improvement on MSIS-29 physical(ES:0.65))
Freeman et al. [26]	in	ACT	MD: patient tailored; towards functional goals	3	?	PT:2; OT: 1; + MD	135	ACT	FIMmotor; (EDSS)	*improvement in comparison with control on FIM motor (selfcare; sphincter; transfer)*(ES:0.21)*; (not on EDSS)
Khan et al. [33]	in/out	ACT	MD:individual, achievable functional goal oriented	7^#^	in: 5; out: 2-3	in:3; out2	in: 180; out:60	ACT	FIMmotor	*difference between groups on FIM motor *(ES:1.13)* and subscales (self care; sphincter; transfers); (not on MSIS *(ES:0.23)* or GHQ)
Mark et al. [27]	out	ACT	CIMT	2-10	?	1	180 (30 h total)	ACT	WMFT; MAL	*improvement MAL (ES2.66), WMFT functional ability (1.17); not on WMFT performance time(ES:0.91)
Patti et al. [28]	out	ACT	comprehensive individualised goal-oriented program	6^##^	6	?	?	ACT (+FU)	ACT:FIMmotor; (FU:EDSS; FSS)	*difference between groups on FIM mot (ES:0.78) and subscales; not on EDSS or FSS
Jones et al. [29]	in	FU + ACT	PT and OT: promoting normal posture and movement (weight baering, approximation,..), stabilisation, equipment, damping and weighting	8 days	7	2	60	FU + ACT	ACT:NPI, JTHF, (FU:FSS)	*difference between groups on NPI and on 4 of 20 JTHF items; not on FSS (FU)
Mathiowetz et al. [30]	in	FU + ACT	MD: OT: compensatory strategies, adapted equipment, conserve enerergy; hand strength; PT:mobility, endurance, strength, streching	5-7	7	?	?	ACT	RIC-FAC	*improvement on all subscales (transfer, toileting, feeding, grooming, dressing upper body, dressing lower body) before-after (ES:0.33-1.19)(also after-follow-up (ES:0.52-1.35), except for feeding)(ES:0.23)
Storr et al. [31]	in	ACT + FU	MD: PT:individualised: joint mobilisation, stretching, relaxtion, balance, coordination, ambulation, hydro-, hypotherapy; OT	3-5	PT:4–5: OT: 3; self training 5	?	PT: 45; OT 30; zelf training 30	ACT (FU)	ACT:GNDS, 9HPT; (FU:EDSS, MSIS)	no *difference between groups on any of outcome measures: 9HPT(ES: 0.01); GNDS(ES 0.08)
Vikman et al. [32]	out	FU + ACT	MD: standard inpatients; PT: group session: strenght, mobility, aquatic, balance; OT: hand therapy	3	ind: 5PT, 3OT; group: 5 PT, 5OT	ind: 1PT,1OT; group: 3PT, 1OT (handfunction)	170	ACT + FU	ACT:BBT, 9HPT, BI, MSFC; FU: Grip strength	A and B:* improvement in MSFC (ES:0.27); and on some of subscales of BBT (ES:0.16) and 9HPT(ES:0.1); not * on BI (ES:0.03) or grip strength (ES:0.08); A* on SF-36

**ACT** (activity); **BBT** (Block and Box Test); **BI** (Barthel Index); **CIMT** (Constraint Induced Movement Therapy); **ES** (Effect Sizes calculated); **ES** (Effect Sizes reported by authors); **ex** (exercises); **FIM** (Functional Independence Measure); **FSS** (Functional System Scale); **FU** (Function); **GHQ** (General Health Questionnaire); **GNDS** (Guy’s Neurological Disability Scale); **In** (inpatient); **ind** (individual); **JTHF** (Jebson Test for Hand Function); **LE** (Lower Extremities); **MAL** (Motor Activity Log); **MD** (Multidisciplinary); **MSFC** (Multiple Sclerosis Functional Composite); **MSIS** (Multiple Sclerosis Impairment Scale); **MSIS-29** (Multiple Sclerosis Impact Scale); **NPI** (Northwick Park ADL Index); **OT** (Occupational Therapy); **out** (outpatients); **PRE** (Progressive Resistance Exercises); **PT** (Physiotherapy); rep (repetitions); **Ric-Fac** (Rehabilitation Institute of Chicago-Functional Assessment Scale); **RM** (Repetitive Maximum); **SET** (Setting); **UE** (Upper Extremities); **WMFT** (Wolf Motor Function Test); **9HPT** (Nine Hole Peg Test); * (significant); ^#^ (plus maintenance home program); ^##^ (plus 6 weeks home program).

Three studies focused specifically on the ICF body function level training [21,22,24,25], two of which only included persons with mild to moderate MS [22,25]. Romberg et al. applied strength and endurance training [21,22] and Taylor et al. focussed on strength training [25]. The submaximal aquatic exercise program applied in Gehlsen et al. was aimed to increase endurance and strength, although the freestyle swimming part of the training was categorised as a complex functional training [24].

Four studies focussed on activity level [26-28,33] and 4 studies combined ICF body function and ICF activity level training [29-32]. These 8 studies included persons with only moderate (n = 2), both moderate and severe (n = 3), or mild to severe (n = 3) MS.

Multidisciplinary rehabilitation was described in 7 studies, in 5 of which the upper extremity intervention was integrated in the total rehabilitation approach [26,28,31,33]. In the other 2 studies, more specific attention was paid to the upper extremity in combination with self-care [30] or trunk stability [29]. The study of Mark et al. described a constraint induced movement therapy focusing specifically on rehabilitation of the arm and hand [27].

With regard to the rehabilitation setting, 4 studies described an inpatient intervention [26,29-31], 5 studies an outpatient intervention [24,27,28,32,33] and in 2 studies a short inpatient intervention was followed by an outpatient intervention [22,33].

#### Training components

In Table 4 the training components of the interventions used in the included studies are presented. The studies included contained between 3 and 12 training components with a mean of 5.5 (SD 2.8) training components used in the different studies. The kappa score between the two raters for the training components was 0.8 (SD 0.2), which was considered a substantial agreement [23]. A significant correlation of 0.67 (Spearman rho)(p < 0.05) was found between the number of components used in a training and the largest effect sizes of the measurement instrument used. The components that were most frequently used were ‘functional movement’ and ‘client-centred’. The components ‘feedback’, ‘random practice’ and ‘distribution-based practice’ were never used. In all 3 studies that focused on the ICF body function level the component ‘exercise progression’ was used. This component was never used in the programmes on the ICF activity level or combined level, with the exception of the study of Mark et al. [27]. The latter study contained the most training components (n = 12). The study of Mark et al. was the only one of the studies using training on the ICF activity level or combined level, that applied the components ‘customised training load’, ‘exercise progression’ and ‘overload’. It was also the only study of the 11 studies included that applied the components ‘context specificity’ and ‘exercise variety’ [27].

**Table 4:** training components

**References**	**Str**	**End**	**Mob**	**Basic**	**Comp**	**FuM**	**Goal**	**CC**	**O/O**	**RO**	**CS**	**Pro**	**Var**	**FB**	**MMP**	**TS**	**CTL**	**Ran**	**Distr**	**Bim**	**Total**
Gehlsen et al. [24]	0	1	0	0	1	1	0	0	0	0	0	1	0	0	1	1	1	0	0	1	8
Romberg et al. [21,22]	1	1	0	0	0	0	0	0	0	0	0	1	0	0	0	0	0	0	0	0	3
Taylor et al. [25]	1	0	0	0	0	0	0	0	1	0	0	1	0	0	0	0	0	0	0	1	4
Freeman et al. [26]	0	0	0	0	0	1	1	1	0	0	0	0	0	0	0	0	0	0	0	0	3
Khan et al. [33]	0	0	0	0	0	1	1	1	0	0	0	0	0	0	1	0	0	0	0	0	4
Mark et al. [27]	0	0	0	0	1	1	0	1	1	1	1	1	1	0	1	1	1	0	0	1	12
Patti et al. [28]	0	0	0	0	1	1	0	1	0	0	0	0	0	0	0	0	0	0	0	1	4
Jones et al. [29]	1	1	0	1	0	1	0	1	0	1	0	0	0	0	0	0	0	0	0	0	6
Mathiowetz et al. [30]	1	1	1	1	1	1	0	1	0	0	0	0	0	0	1	0	0	0	0	0	8
Storr et al. [31]	0	0	1	0	1	0	0	0	0	0	0	0	0	0	1	1	0	0	0	1	5
Vikman et al. [32]	1	0	1	1	0	0	0	0	0	0	0	0	0	0	0	0	0	0	0	0	3
Frequencies	5	4	3	3	5	7	2	6	2	2	1	4	1	0	5	3	2	0	0	5	60

Bim (bimanual tasks included); CC (client-centred); Comp (complex); CS (context specific); CTL (customized training load); Distr (distribution based practice); End (endurance); FB (feedback); FuM (functional movement); Goal (clear functional goal); Mob (mobility/stretching); MMP (multiple movement planes); O/O (overload/overlearning); Pro (exercise progression); Ran (random practice); RO (real objects); Str (strength); TS (total skill); Var (exercise variability).

#### Outcome: Training effect

The studies in which training of the upper extremity was focused on the ICF body function level [22,24,25], demonstrated that it was possible to perform strength and endurance training and that this kind of training may lead to a significant improvement of the upper extremity on the ICF body function level [22,24,25](with moderate to large ES on the ICF body function level outcome measures in the study of Gehlsen et al. and Taylor et al.). The studies reporting training on activity level revealed a significant improvement on the ICF activity level outcome measures in comparison with the control group [26,28,33]. A large ES on the FIMmot was found in the study of Patti, large ES were reported by Kahn et al. and moderate ES by Freeman et al.. Furthermore, a significant improvement on activity level outcome within the experimental group was reported in the study of Mark, demonstrated by large ES.

The results of the studies in which training at activity and body function level was combined, were not consistent. The study of Storr et al. did not reveal significant differences between the groups in any of the outcome measures and small effect sizes were found [31]. The study of Jones et al. demonstrated a significant difference on some of the outcome measures at the activity level in favour of the experimental group [29]. Furthermore, a significant improvement on the ICF activity level outcome measures (RIC-FAC) within the experimental group was reported in the study of Mathiowetz et al., demonstrated by moderate to large ES for most of the subscales [30]. In the study of Vikman et al. a significant improvement was described on some, but not all, of the ICF activity level outcomes measures. No significant improvement on the ICF body function level outcome measures (grip strength) was found [32]. In the latter study all effect sizes were rather small.

## Discussion

The aim of this study was to investigate the outcome of motor training programs of arm and hand functioning (on the ICF body function and the ICF activity level) and their training components in persons with MS.

In general, it can be concluded that there are a limited number of studies on upper extremity training in persons with MS displaying a wide variety regarding patient characteristics, interventions and outcome measures. Apart from the study of Storr et al. [31], all studies demonstrate an improvement in upper extremity at the ICF body function and/or the ICF activity level in MS patients with different degrees of severity. This conclusion has a level of 2 according to the Dutch CBO guidelines (see Additional file 1: Appendix A) [34].

It was remarkable that only one (pilot) study [27] was exclusively dedicated to the upper extremity. Given the importance of the upper extremity functioning, a discrepancy exists between clinical relevance and research; i.e. the clinical experience that may be present in the field, is not explicitly reported or supported by evidence.

With regard to the outcome of the 11 studies included, it can be concluded that most of the studies reported an improvement at the same ICF level the training was focused on, in which a transfer towards improvement on other ICF levels did not occur, except in study Taylor et al. [25]. For example, in the study of Romberg et al. a body function level training resulted in an improvement at the ICF body function level, but not in an improvement on the FIM (the ICF activity level) [21,22]. These findings support the importance of the specificity of the training, which was also emphasised the review of Van Peppen et al. for stroke [4] and Spooren et al. for SCI [3]. In clinical practice, it means that when the training of upper extremity, the specific task, that the patient would like to learn, should be incorporated. This may improve rehabilitation outcome and is in agreement with principles of motor learning [36].

With regard to the number of training components, a significant correlation was found between the number of components used in the training and the effect sizes. This is in contrast with the findings in the study of Timmermans et al. [5] who found no significant correlation. On the one hand, the significant correlation in the present study may be attributed to the fact that the study of Mark et al. [27] incorporated most training components (12) and demonstrated largest effect sizes. When omitting the study of Mark et al., the correlation between the number of components used in the training and the effect sizes was still 0.57 (Spearman rho), but it failed to be statistically significant. On the other hand, incorporating markedly different training components may indeed improve rehabilitation outcome. However, besides the number of training components, the content of the training components may play an important role on effect size as has been suggested by Timmermans et al. [5] and which will be explained in the section below.

In the present review, all the 3 studies with the ICF body function level training [22,24,25] included the component ‘exercise progression’, which was not included in the studies with training at the ICF activity level or combined level, except in the study of Mark et al. It is expected that incorporating ‘exercise progression’ into an ICF activity level training may result in better rehabilitation outcome.

The studies with training on the ICF body function level did not include the component ‘client-centred’ [22,24,25]. Neither did two studies with training at combined level include the component ‘client-centred’ i.e. the study of Storr [31] and the study of Vikman [32]. The latter studies [31,32] reported low effect sizes and mixed findings regarding the level of improvement. Even more, the study of Storr et al. was the only study that that did not reveal any positive finding [31]. They argued that his may be attributed to different factors, e.g. the choice of the outcome measures, the population that they included or the design. However, they also suggested that it might be due to the fact that the patients in their study were admitted without specific rehabilitation needs, thereby preventing the treatment from being dedicated towards patients’ individual needs. The latter argumentation is corroborated by earlier research results reporting that client-centred care and individual goal setting increases patients’ motivation [37,38], that patients tend to be more pro-active [39] and that an individualised rehabilitation program, tailored to patient’s self-selected goals, may improve rehabilitation outcome [40]. Indeed, in the 6 studies of the present review in which the component ‘client-centred’ was identified [26-30,33], a significant improvement was reported, with moderate to large ES in 5 of them [26-28,30,33].

In the review of Timmermans et al., targeting stroke trials, the component ‘client-centred’ was associated with low effect sizes [5]. This could be due to the fact that this treatment focused on specific goals, which are not always measurable with standardised outcome measures. Furthermore, it could be due to the fact that therapists could not sufficiently control training parameters like intensity and load during the client-centred home trainings [5]. However, it may be possible that combining a client-centred approach with components related to motor learning, like context specificity and exercise variety, and principle to grade and progress the client-centred training in which training parameters are well controlled, result in better outcome after training and at follow-up. The latter is supported by the studies of Spooren et al. who applied a task-oriented client-centred training program (ToCUEST) in SCI [18,41] and Timmermans et al. who applied a client-centred technology supported task-oriented training program (T-TOAT) in stroke [42]. Both programs combined the client-centred component with the explicit use of various training components, based on motor learning and training physiology, and demonstrated an improvement in upper extremity functioning [41,42].

In the present review, it was remarkable that none of the studies included applied the components ‘feedback’, ‘distribution based practice’ or ‘random practice’ to train the upper extremity in MS. Yet in the review regarding task-specific training programmes in stroke by Timmermans et al. [5], it was concluded that’feedback’ and ‘distribution based practice’ were associated with higher effect sizes post intervention and ‘random practice’ was associated with the largest effect sizes at follow-up [5] . Given the importance of these components in the literature of motor learning [36] and previous research [5], it would be interesting to investigate the influence of these components on effect sizes in upper extremity training programs for MS.

The studies with the highest effect sizes in the present review were the studies of Mark et al. [27] and Kahn et al. [33]. Both studies used an intensive training program directed towards patient’s individual goals. The study of Mark et al. used a Constrained Induced Movement Therapy program in which an intensive well structured training program was applied incorporating many principles of training physiology and motor learning and which was directed towards individual goals. High effect size may be attributed to the intensity and the content of the training, as well as to the effects to overcome learned non-use of the impaired upper limb in patients with MS. The training program used in the study of Kahn et al. was also an intensive program aimed at improving activity and participation and directed to individual functional goals. It consisted of an intensive inpatient and outpatient program and was followed by a maintenance home program. The high effect sizes could again be attributed to the intensive individual goal directed training with longer duration. Kahn et al. mentioned also that an adequate cognition level is an important factor in individual goal setting.

### Methodological considerations

Patient characteristics may influence effect sizes. However, post-hoc analyses showed that the most important patient characteristics such as EDSS-score, type of MS and age were equally distributed over the interventions with low, medium and high effect sizes*.* It should be noted that, as far as reported in the studies included, studies focussing on training at the ICF body function level included patients with mild to moderate MS with an EDSS score from 0–6.5. Therefore, it may not be concluded that training at the ICF body function level leads to improvement in all patients with MS. The studies focussing on training at the ICF activity level or combined level included MS patients with all degrees of severity (EDSS score ranging from 0–9). Furthermore, in persons with MS, fluctuations in impairment may also influence the outcome of training programs. All the studies presented in this review excluded patients with exacerbation in which most of the studies included stable MS patients, i.e. mostly 3 months after the last exacerbation. Post hoc analysis showed that the time since the last exacerbation was equally distributed over the intervention with low, medium and high effect sizes. Therefore, one could argue that the training effects described in the different studies were not influenced by the fluctuations. However, clinicians should take into account the considerations with regard to the patient characteristic and the fluctuation of the impairment when composing a training program.

Although a meta-analyses correlating the components with effect sizes would give more definite information, this was impossible to do in the present review. From one study, no effect sizes could be retrieved. From two other studies, effect sizes reported in the paper were taken, which may be different than the Hedges’g effect sizes, making a meta-analysis difficult to interpret. However, such analysis should be performed in future research.

The Van Tulder score was used to gauge the methodological quality of the studies included. Seven of the eleven studies obtained a score of 9.5 or more, which was the quality cut-off point of the Van Tulder score. However, all the 11 studies were reported and analysed in the present study. Firstly, because the aim of the present review was to provide a comprehensive overview of the existing studies with regard to upper extremity motor programs. Secondly, because the Van Tulder score was mainly designed for RCT’s and controlled clinical trials and 5 of the studies included were case series [24,25,30,32] or a pilot study [27] resulting in a 0-score on the items related to RCT or Controlled Clinical Trial. Additionally, the considerations and conclusions drawn in this review were based on a group of studies displaying similar characteristics including studies with a good Van Tulder score.

## Conclusion

This review revealed that a limited number of training programs are available regarding the upper extremity in MS that are supported with RCT or CCT evidence. Apart from 1 study, all studies demonstrate an improvement in upper extremity at the ICF body function and/or activity level in medical stable MS patients with different degrees of severity. The results demonstrate the importance of the specificity of the training and the inclusion of the training components ‘client-centred’ and ‘exercise progression’. Incorporating more training components with regard to motor learning and training physiology may lead to improved rehabilitation outcome, although more research is needed to corroborate this. The lack of the training components ‘feedback’, ‘distribution based practice’ or ‘random practice’ was remarkable. Given the importance attributed to these components in stroke, the use of these components to optimise training should be further explored in future research.

## Abbreviations

MS: Multiple sclerosis; (C)-SCI: (Cervical) Spinal cord injury; ES: Effect Sizes; SD: Standard deviation; RCT: Randomised controlled trial; EDSS: Expanded disability status scale; ICF: International Classification of Functioning Disability and health; CBO guidelines: Central guidance organisation for quality in healthcare.

## Competing interest

Authors declare that they have no competing interest.

## Authors’ contributions

AS carried out the main contribution to the conception, design, acquisition of data, analysis and interpretation of data and had the main contribution in writing the manuscript. AT made a substantial contribution to the conception, design, acquisition of data, analysis and interpretation of data. She contributed to the writing of the manuscript and revised it critically for important intellectual content. HS made a substantial contribution to revising the manuscript critically for important intellectual content. All authors read and approved the final manuscript to be published.

## Pre-publication history

The pre-publication history for this paper can be accessed here:

http://www.biomedcentral.com/1471-2377/12/49/prepub

## Supplementary Material

Additional file 1 **Appendix A.** Levels of evidence according to the Dutch CBO guidelines. Click here for file

Additional file 2 **Appendix B.** Van tulder score. Click here for file
