# Supplementary material for: Motor training programs of arm and hand in patients with MS according to different levels of the ICF: a systematic review
Source: BMC Neurol. 2012 Jul 2;12:49. doi: 10.1186/1471-2377-12-49 (PMC3527200; doi:10.1186/1471-2377-12-49)
Supplement: Additional file 1 — Appendix A. Levels of evidence according to the Dutch CBO guidelines. [file 1471-2377-12-49-S1.doc]

Appendix A: Levels of evidence according to the Dutch CBO guidelines

| A1 | Systematic review containing at least 2 independent trials of level A2 |
| --- | --- |
| A2 | Randomized comparative double-blind study of good quality and sufficient size |
| B | Comparative trials, but not all characteristic of A2 (also patient control studies and cohort studies) |
| C | Non-comparative trials |
| D | Expert opinion |
| Level of conclusion according to the Dutch CBO guidelines | |
|  | Conclusion based on |
| 1 | Research on level A1 of at least 2 independent trials of level A2 |
| 2 | 1 trial of level A2 of at least 2 independent trials of level B |
| 3 | 1 trial of level B or C |
| 4 | Expert opinion |
