# Supplementary material for: Motor training programs of arm and hand in patients with MS according to different levels of the ICF: a systematic review
Source: BMC Neurol. 2012 Jul 2;12:49. doi: 10.1186/1471-2377-12-49 (PMC3527200; doi:10.1186/1471-2377-12-49)
Supplement: Additional file 2 — Appendix B. Van tulder score. [file 1471-2377-12-49-S2.doc]

Appendix B: Van tulder score

| **References** | **A** | **B1** | **B2** | **C** | **D** | **E** | **F** | **G** | **H** | **I** | **J** | **K** | **L** | **M1** | **M2** | **N** | **O** | **P** | **Q** | **TOTAL** |
| --- | --- | --- | --- | --- | --- | --- | --- | --- | --- | --- | --- | --- | --- | --- | --- | --- | --- | --- | --- | --- |
| Gehlsen et al. [31] | 1 | 0 | 0 | 0 | 1 | 0 | 1 | 0 | 0 | 0 | 1 | 0 | 0 | 1 | 0 | 0 | 1 | 0 | 1 | 7 |
| Romberg et al. [21,22] | 1 | 0 | 0 | 1 | 1 | 1 | 0 | 0 | 1 | 0 | 0 | 1 | 1 | 1 | 1 | 1 | 1 | 1 | 1 | 13 |
| Taylor et al. [30] | 1 | 0 | 0 | 0 | 1 | 0 | 1 | 1 | 0 | 0 | 1 | 1 | 1 | 1 | 0 | 0 | 1 | 1 | 1 | 11 |
| Freeman et al. [26] | 1 | 0 | 0 | 1 | 0 | 0 | 1 | 0 | 0 | 0 | 1 | 1 | 1 | 1 | 0 | 1 | 1 | 0 | 1 | 10 |
| Khan et al. [10] | 1 | 1 | 1 | 1 | 1 | 1 | 0 | 1 | 0 | 1 | 1 | 1 | 1 | 0 | 1 | 1 | 1 | 1 | 1 | 16 |
| Mark et al. [32] | 1 | 0 | 0 | 0 | 1 | 0 | 0 | 0 | 0 | 1 | 1 | 1 | 1 | 1 | 0 | 0 | 1 | 0 | 1 | 9 |
| Patti et al. [24] | 1 | 1 | 1 | 1 | 1 | 0 | 0 | 0 | 0 | 1 | 1 | 1 | 1 | 1 | 1 | 1 | 1 | 1 | 1 | 15 |
| Jones et al. [35] | 1 | 0 | 0 | 1 | 1 | 0 | 0 | 0 | 0 | 0 | 1 | 0 | 0 | 1 | 0 | 1 | 1 | 0 | 0 | 7 |
| Mathiowetz et al. [34] | 1 | 0 | 0 | 0 | 1 | 0 | 0 | 0 | 0 | 0 | 1 | 1 | 1 | 1 | 0 | 0 | 1 | 1 | 1 | 9 |
| Storr et al. [27] | 1 | 0 | 0 | 0 | 1 | 1 | 0 | 0 | 1 | 1 | 1 | 1 | 1 | 1 | 0 | 1 | 1 | 0 | 1 | 12 |
| Vikman et al. [33] | 1 | 0 | 0 | 0 | 1 | 0 | 1 | 0 | 0 | 0 | 1 | 1 | 0 | 1 | 0 | 1 | 1 | 1 | 1 | 10 |
| **Frequencies** | 11 | 2 | 2 | 5 | 10 | 3 | 4 | 2 | 2 | 4 | 10 | 9 | 8 | 10 | 3 | 7 | 11 | 6 | 10 |  |

**A** (eligibility criteria); **B1** (randomised); **B2** (treatment allocation concealed); **C** (groups similar at baseline); **D** (intervention explicitly described); **E** (care provider blinded for intervention); **F** (co-intervention avoided or comparable); **G** (compliance); **H** (patient blinded); **I** (outcome assessor blinded); **J** (outcome measures relevant); **K** (adverse effect described); **L** (withdrawal acceptable); **M1** (short-term follow-up); **M2** (long-term follow-up); **N** (timing assessment comparable); **O** (sample size described); **P** (intention-to-treat analysis); **Q** (measures of variability described)
